# The Health of the People and the Ministry of Health

**Published:** 1918-11-02

**Authors:** 


### THE HEALTH OF THE PEOPLE AND THE MINISTRY OF HEALTH.

(FROM A CORRESPONDENT.)

THAT the demands of the public for a Ministry of Health will soon materialise is probable. The Prime Minister's statement, based on proofs supplied by the Ministry of National Service, that we are a nation of C3 men calls urgently for war on the causes of this imperfection in our national health.

The causes of this low standard of health are mainly based on the conditions under which the masses toil for their livelihood. Workers on the land are worn out at forty from strain and exposure, and workers in towns are worn out at forty by overwork, underfeeding, and impure air. The results of the survey of the national physique by the National Service Medical Boards have been appalling. Professor Arthur Keith, writing in the *Observer* of October 20, 1918, fixes as a standard of physical fitness of a healthy population the following proportions per 1,000:—

| | |
|---|---|
| Grade 1.—Good physique ... ... ... | 700 |
| „ 2.—Fair physique ... ... ... | 200 |
| „ 3.—Bad physique ... ... ... | 75 |
| „ 4.—Unfit for any of the duties of a soldier ... ... ... ... | 25 |

and contrasts a typical result of grading 1,000 men drawn at the present time from one of our large industrial towns.

| | |
|---|---|
| Grade 1 ... ... ... ... ... ... | 190 |
| „ 2 ... ... ... ... ... ... | 270 |
| „ 3 ... ... ... ... ... ... | 410 |
| „ 4 ... ... ... ... ... ... | 130 |

Even the results obtained from trades which had been exempted in the earlier years of the war are disappointing, a result such as the following being only too common:—

| | |
|---|---|
| Grade 1 ... ... ... ... ... ... | 270 |
| „ 2 ... ... ... ... ... ... | 152 |
| „ 3 ... ... ... ... ... ... | 158 |
| „ 4 ... ... ... ... ... ... | 420 |

Over four companies of this possible battalion were useless for any military purpose.

### A HEALTHY ENVIRONMENT.

For the maintenance of health the fundamental requirements are: proper housing—rooms which can be ventilated and warmed, and which are not over-crowded—suitable cooking and sanitary arrangements, baths, a pure and sufficient water supply, good food, sufficient clothing, and hours of work, under healthy conditions, sufficiently short to allow time for recreation and sleep. This programme implies expenditure, therefore sufficient money must be obtainable by work and by universal insurance.

Worry, domestic or business, exerts an enormous influence on the public health, a well-managed community, like a well-managed business, should have few worries. The supreme necessity, therefore, is the provision and maintenance of healthy environment for every individual, irrespective of class, age, or sex. The prevention of disease must be accorded priority over all other medical needs.

The prevention of oral sepsis is a burning question: a regular system of dental inspection and treatment in the schools would have far-reaching results. The mouth and throat are the portals of entrance of such diseases as rheumatic fever, diphtheria, scarlet fever, and measles; and pyorrhœa, with its consequent toxæmia, reacting on digestion, nutrition, and the purity of the blood, is responsible for much preventable disease. Neglect of the teeth is almost universal in Britain; it is most exceptional to find healthy teeth in a single individual outside the educated classes. This is in marked contrast to the conditions prevailing in the Dominions and in America, where the teaching is thorough, the practice aseptic, and the results enduring.

In addition to improved hospitals the service of the public requires improved doctors, and this involves a revolution in medical education, in the framing of which Sir George Newman's recent memorandum on medical education to the President of the Board of Education should act as a useful guide, its keynote is "co-ordination," of the several subjects taught in the class-rooms and of clinical practice in the wards, and afterwards in public and private practice. The relation of voluntary hospitals to the Ministry of Health was under the consideration of a meeting of the members of the British Hospitals Association which was fully reported in THE HOSPITAL of October 26, page 79. The Bill setting up a Ministry of Health, as originally drafted, has now been withdrawn pending the discussion of a new draft entitled the Ministry of Health and Local Government Bill.

Until the issue of the new Bill it is not possible to discuss its provisions or the effect which it may have, directly or indirectly, on the voluntary system of hospitals and their administration. The people in these islands realise that under the voluntary system the patient is the first consideration, and there is a practically unanimous feeling that the voluntary systems of hospitals must be maintained and strengthened materially in many ways. If the new Bill attempts to set the clock back and cripple the prospects of an adequate Ministry of Health for this country by tacking on to it the non-medical side of the Poor Law, all such attempts must be defeated in the interests of the public health.

